# The Hospital. Nursing Section

**Published:** 1907-01-05

**Authors:** 


### The Hospital.

### Nursing Section.

Contributions for "THE HOSPITAL," should be addressed to the EDITOR, "THE HOSPITAL" NURSING SECTION, 28 & 29 Southampton Street, Strand, London, W.C.

NO. 1,060.—VOL. XLI. SATURDAY, JANUARY 5, 1907.

### Notes on News from the Nursing World.

### THE KING AND DISTRICT NURSING.

THE interest which the King takes in the progress of district nursing is manifested in a practical manner by the gift which his Majesty has just made of £20 from the Windsor Castle State Apartments Fund to the Slough Nursing Fund. In addition to its intrinsic value the donation may be regarded as the personal testimony of the Sovereign to the excellent work done by the trained nurses employed by the Fund to attend the poor, who form a very numerous proportion of the inhabitants of Slough.

### ROYAL NATIONAL PENSION FUND FOR NURSES.

THE year just closed has been one of unprecedented success for the Pension Fund. In spite of the fact that the work of 1905 showed a huge advance on any previous year, 1906 surpassed all its predecessors. A most notable point is that during the last three months more nurses entered the Fund than in any quarter since its establishment. During the twelve months the sum of £114,000 was received in premiums, and the total income exceeded £155,000. The invested funds have now reached a total considerably over a million sterling. There was a great increase in the amount paid out in annuities, which are now being distributed at the rate of over £16,000 per annum, or £3,000 a year more than in 1905.

### AN INCIDENT IN A POOR-LAW INFIRMARY.

THERE was an inquest at Hackney on Friday on the body of a woman who died at Hackney Union Infirmary on Boxing Day, and it was stated that she fell out of bed during the night of December 20 while the nurse was out of the ward. Nurse Leys, who gave evidence, said that she had charge of a ward containing thirty patients, and was on duty from 7.30 P.M. to 8 A.M., and that she had one day off a fortnight. As a matter of fact she is also off duty from 8.30 till 10.30 A.M. each day, on Sunday till 10 P.M., and once each five weeks from 8.30 A.M. till 1 P.M. next day, when she can spend the night at her home or with her friends. Moreover, we are informed that if there are acute patients in the wards an extra nurse is always put on to assist. The accident occurred at 5.45 A.M., when Nurse Leys was preparing the patients' breakfast in the kitchen close to the ward. Night nurses are obliged to leave the ward for this purpose, and also to prepare dressings. Miss Leys has been on the staff since November 11, 1904, and is 27 years of age; though we are pleased to notice that the juryman who asked the nurse how old she was was promptly censured by the coroner, who remarked that it was no business of his, the nurse being evidently an able-bodied person. The jury added to their verdict of "accidental death" a rider "that no nurse should be kept on duty all night in a ward with thirty beds without assistance, or that the hours should be shortened." The hours are certainly long, and as it is impossible to prepare breakfast for thirty patients and to get their dressings ready without spending considerable time out of the ward, we think that, in the indispensable absence of the charge nurse, there should be a probationer available. As we have so often urged, no ward containing a number of sick persons should be left without a nurse.

### POOR-LAW PROBATIONERS AND MIDWIFERY.

A VERY interesting point is under the consideration of the Staff Committee of the Hull Guardians. It has been proposed, at the instance of the medical officer, that the sum of £10 shall be spent in instructing five probationers in midwifery, so as to pass the qualifying examination. Objection has, however, been taken on the ground that four of the five probationers are leaving shortly, and it is urged that in these circumstances they ought not to sanction the expenditure of the ratepayers' money. The Guardians who favour the proposal rejoin that the probationers in question have been capital workers, and that if they are sent out in every respect qualified, the best class of candidates will be attracted when vacancies on the staff are announced. We hope, in their own interest, that the Guardians will decide to adopt the recommendation. If it be essential to avoid the waste of the ratepayers' money, it is also advisable to offer every reasonable inducement to young women to enter the Poor-law nursing service; and instruction in midwifery at the cost of £2 a head may fairly be included in this category.

### THE REGIMENT AND THE GENERAL ARMY.

AT the annual meeting of the Cheltenham District Nursing Association the Chairman of the Executive Committee formally announced that it was affiliated to the Queen's Jubilee Institute. As he anticipated that people would ask why an organisation nine years older than the Institute itself should have considered it necessary to take that course, he said that a time came when it was recognised that the regiment must be enrolled in the general army. It was a bad thing to be isolated, he continued, and the work of the Queen's nurses extended over the whole country, and was conducted on rules practically identical with those which the Cheltenham Association had invented. He affirmed that a great stimulus was afforded by connection with a large body like that represented by the Jubilee Institute, and he was confident that on every ground advantage would accrue. He especially emphasised the benefit of periodical and independent inspection of the nursing staff. Subsequently, Miss Amy Hughes delivered an address, in which she expressed the pleasure it had given her to revisit a town which was one of the pioneers in the great work of district nursing; and other speakers intimated their satisfaction that the affiliation had been arranged.

### A PROBATIONER LOSES HER SIGHT.

A deplorable event, illustrating the risks which nurses run in the discharge of their duty, took place the other day at West Ham Poor-law Infirmary. A patient suffering from typhoid fever spat in the eye of a probationer in attendance. Two specialists were called in at once, but the sight of one eye has been destroyed and that of the other impaired. The guardians have assured the probationer that, "so long as she behaves herself," she will be employed by them; and that, we suppose, is the only consolation they are able to offer the unfortunate girl, for whom everybody will, we are sure, feel the utmost sympathy.

### A BROKEN AGREEMENT.

The Chelmsford Guardians recently appointed as charge nurse at the Poor-law Infirmary a nurse from Sheffield. She appeared before the Board in person, was unanimously elected, received her expenses, and agreed to commence her duties after Christmas. Four days later she sent a telegram saying that, "owing to unforeseen circumstances," she was unable to take up the appointment. We think that the Guardians, who are very indignant at the way in which she has behaved, have cause for complaint. They have been put to needless expense, and have rejected applicants not less suitable who, it appears, are now engaged elsewhere. A nurse should never accept an appointment unless she has fully made up her mind to carry out her agreement. We presume that the nurse who broke her agreement has since returned her expenses.

### FATAL FALL FROM A TRAM-CAR.

Nurses in all countries so freely use tram-cars that the sad case of a New Zealand nurse merits attention. This nurse, who was attached to a Nurses' Institute in Auckland, was hurrying to attend an urgent case, and in her haste got out of the tram-car while it was in motion. Such, at least, was the conclusion formed at the inquest, though it was admitted to be possible that her fall might have been caused by the jolting of the car while she waited on the step. The result, anyhow, was that her head struck the road with great force, the base of the skull being terribly fractured. Unfortunately it was necessary to have the funeral before any of the relatives of the deceased could arrange to be present, but many nurses followed her remains to the grave, and numerous wreaths were sent. It is very important that a nurse should lose no time in proceeding to a case, especially if it be described as urgent; but her first obligation is to take care of herself and see that she does not meet with an accident on the way. No one should ever leave a tram-car until it has come to a standstill.

### A HAPPY SITUATION AT SUNDERLAND.

The nurses attached to the Sunderland District Nursing Association are to be congratulated upon the important extensions which have been made to the home. Lady Londonderry, who performed the task of declaring the premises open, affirmed that the subscription list and the number of cases nursed, which was nearly 1,200, indicates that the work of the Association is thoroughly appreciated and understood in Sunderland. There is generally no difficulty in attending an adequate number of cases, but when, in addition to this, the list of subscribers is all that could be desired, the advantages of district nursing to a community do not need to be urged; they have been clearly apprehended.

### NEGLECTING TO NURSE THE CHILDREN.

The Lincoln Guardians have decided to add to the number of the nursing staff attached to the infirmary wards. When the subject was under discussion a member of the Ladies' Nursing Committee, to whom the question had been referred, mentioned that the medical officer said it was absolutely necessary that another nurse should be appointed, and he added that "the most neglected corner was the children's ward." Such a statement as this should not have been possible. The children, if simply because of their tender years, should be the last to be neglected; and we hope, for the credit of the Lincoln Guardians, that in future their ward will receive adequate attention from the nursing staff.

### COTTAGE HOSPITALS AND STAFF NURSES.

We mentioned a fortnight ago the large number of applications received for the post of matron of a cottage hospital. In contrast to this the authorities of Redhill Cottage Hospital advertised the other day for a staff nurse, and there was not a single candidate for the post. Of course it is the desire of most nurses to become a matron, and the appointment of staff nurse in a small institution does not seem to offer much scope for ambition. But it affords plenty of opportunities for useful work, and at Redhill Cottage Hospital, which contains thirty-one beds—nineteen of these being generally occupied—the three staff nurses assist in the training of the three probationers.

### AN ASSISTANT SUPERINTENDENT FOR BIRKENHEAD INFIRMARY.

The appointment of Miss Copeland as assistant superintendent nurse and house sister at Birkenhead Union Infirmary, which we announce in another column, marks a step in advance, that was not adopted without opposition. Miss Copeland, who was trained at Birkenhead Infirmary and has been charge nurse there for three years, was chosen from a number of applicants, and she is the first occupant of the office to which she has been elected. The creation of the office was protested against by a guardian on the ground that it was contrary to the rules and regulations, but he ultimately gave way and expressed himself satisfied that the superintendent nurse needs the help which she is now to receive. We have no doubt that the nursing staff and the patients, as well as the superintendent nurse, will profit by the new arrangement.

### RESIGNATION OF A MATRON.

The post of lady superintendent of nursing and of housekeeping at the Queen's Hospital, Birmingham, has become vacant by the resignation of Miss C. Elkington. Applications for the post will be received by the Committee of Selection up to January 31. The commencing salary is £100 a year.

### NURSES' AND OTHER ENTERTAINMENTS AT GUY'S HOSPITAL.

An excellent concert was given on Friday last in the Physiological Theatre at Guy's Hospital. Mr. Seymour Hicks and Miss Ellaline Terriss took part in the performance, and charmed the audience with their talent. Sir Alfred Fripp acted as host. Many of the convalescent patients from all parts of the hospital were able to be present. This entertainment was on behalf of "Luke" and "Charity" wards. On Wednesday, Thursday, and Saturday evenings the residents gave most delightful entertainments of both music and theatricals, to which their friends, the medical, surgical, and nursing staffs, were invited. On Friday evening the nurses gave their entertainment in the Home, which was beautifully decorated for the occasion by the nurses. The staff and residents were the guests of the evening. On Tuesday, January 1, the nurses repeated their performance in the Physiological Theatre, at which a silver collection was made for the benefit of the Recreation Society Fund.

### TRAINING IN VICTORIA.

A Conference of Hospital Managers and the Royal Victorian Trained Nurses' Association was held in Melbourne Town Hall on Friday, November 23. Representatives from forty-three hospitals met delegates from the Association to finally arrange various items in the training of nurses. The Queen's Memorial Hospital for Infectious Diseases naturally looks upon itself as the correct and only place capable of giving this special training, while many of the country hospitals are averse to sending their pupils to Melbourne for this certificate when they have infectious wards in their own institutions. The Association accepted the conditions laid down by the hospital managers that where the pupils can give evidence of having nursed a sufficient number of infectious cases, and have passed a special examination in town, the special certificate shall be given. This certificate is only compulsory for those who enter for the Matron's Examination, though private nurses are advised to take it up. The next question was the recognition of hospitals with less than ten daily occupied beds. The Association was very unwilling to recognise any training in such institutions, but strong arguments were brought to bear on the financial difficulty of employing trained nurses; and eventually it was decided to allow them to take pupils for three years, with a compulsory six months at the Women's Hospital, Melbourne, and three months at the Infectious Diseases Hospital.

### EVENING LECTURES TO NURSES.

A series of Lectures by medical men to nurses will be commenced at the Miller Hospital, Greenwich Road, on Tuesday evening, January 8, and continued every Tuesday except March 26, April 2 and 9, until May 28. They will commence at eight o'clock, and are free to all nurses. Application for tickets should be made to the matron of the Miller Hospital.

### GATHERING OF MALE NURSES.

An interesting gathering of male nurses took place at the National Hospital, Bloomsbury, on December 21, to bid farewell to Nurse Suckling, who has completed his training at that institution. Mr. Cope, senior staff nurse, presided, and, having expressed the good wishes of the staff, said he hoped that Mr. Suckling's future would be happy and prosperous. He considered that the old prejudice against male nurses was rapidly giving way, and it was now recognised that the hospital trained and certificated male nurse was a necessity. He urged every nurse present to do his utmost for the credit of the National Hospital, and to strive to raise the standard of nursing.

### THE REGISTRATION FEE IN AUSTRALIA.

At a special meeting of the Royal Victoria Trained Nurses' Association, held on Wednesday, November 21, the question was raised whether a registration fee in one sum of £2 2s. or £3 3s. would not be a more satisfactory arrangement than the 10s. 6d. per annum, which has been, and is, such a trouble to collect. The matter was deferred to the February meeting.

### "THE HOSPITAL" CONVALESCENT FUND.

The Hon. Secretary begs to acknowledge with thanks the receipt of 2s. 6d. from Nurse Barnes-Groom. Annual subscribers are reminded that their kind subscriptions for 1906 should be sent in immediately. The work of this Fund is proceeding steadily, and during 1906 most deserving cases have been helped. Nurses are most generous and kind-hearted to their fellows in distress, and all are therefore appealed to once again to contribute something, however small in amount, to The Hospital Convalescent Fund, and thus ensure that no deserving case brought to its notice shall be neglected for want of funds.

### SHORT ITEMS.

The sale recently held in aid of the Hospital for Invalid Gentlewomen, in Harley Street, realised, we are glad to hear, upwards of £150.—On Monday last an enjoyable evening was spent by the in-patients of the Cancer Hospital, Fulham Road, at an entertainment given by the Lancaster Pierrots for Mrs. Read.

### The Nursing Outlook.

"From magnanimity, all fears above;
From nobler recompense, above applause,
Which owes to man's short outlook all its charm."

### A NOBLE WOMAN.

THE late Baroness Burdett-Coutts at the age of twenty-three suddenly came into possession of a fortune so large as to make her the envy and wonder of her contemporaries. In 1837 millionaires were few in number, and such as there were had their wealth so hampered by claims and restrictions, that few could command large sums, even, for their own purposes. Miss Angela Georgina Burdett as a millionaire at twenty-three, with an abundance of ready money and an immense income for those days, was not unnaturally envied by many. She became the centre to which all sorts of people directed their attention for their own purposes. At this time the position of a woman of wealth, who was young and unmarried, was deemed insecure and indeed unsafe, so that public opinion expected her to select a husband, as the wisest step she could take for her own protection. It required much character and resolution to put aside all such projects, as the late Baroness did, and to resolve to be the mistress of her own fortune and its custodian. It was a great opportunity for the exercise of wisdom and example, for at this time the wise administration of charity was neither understood nor practised. Beset by suitors for her hand, overwhelmed with applications for monetary gifts for all kinds of schemes and individuals, worried by the importunate, and wooed by the designing, the position, in which Miss Angela Burdett found herself, was not only difficult but bewildering. That she should have faced it with courage and displayed marked originality and judgment in the solution of the difficulties presented, must ever make the Baroness' name memorable in English history.

Recognising that the exhibition of great ability was as essential to the wise administration of wealth as to its acquirement, the Baroness sought the best available advice, not only in the management of her business, but in the administration of her charities. Her sympathies were wide and comprehensive. She aimed, especially, at helping the poor to maintain themselves in comfort, to obtain adequate housing accommodation, to secure an abundance of wholesome food at the cheapest rates, and a supply of suitable work at adequate wages, especially in times of depression and difficulty. Her sympathies extended to the poorer citizens of London and of the United Kingdom. Her efforts were not restricted to the metropolis, for she did much to help distressed communities both in Scotland and Ireland. By her schemes of emigration she was the means of rescuing many poor families from poverty, and placing them in a position of comfort and independence. She had great sympathy with the animal kingdom, and was untiring in her efforts to promote the kind treatment of horses and donkeys used in the streets, whilst her devotion to dogs and singing birds caused her to write many excellent letters to the Press which led to legislation, and were followed by improvements in many directions. No animal was too small nor too miserable to win her sympathy, and no scheme for the welfare of poor or down-trodden people, too large to secure her most generous support. She lent her aid to the improvement and extension of education, and was, in fact, the pioneer of technical education in this country, as the history of the Westminster Institute of Technical Training testifies. Without a family of her own, her sympathies were warmly engaged in the service of children, and it was the Baroness who inaugurated the Royal Society for the Prevention of Cruelty to Children. We have but enumerated some of the greater objects which engaged her attention, but throughout her life the Baroness maintained a number of almoners, whose duty it was to provide, adequately, for the needs of a multitude of individual cases of distress or suffering, in which she took the deepest personal interest.

Great as may have been the good accomplished by the generosity by which all her actions were supported, the Baroness Burdett-Coutts' claim to recognition in history will deservedly rest upon the results for good, which have flowed from her example. By her wisdom and spirit she proved the baselessness of the fears that disaster might befall her at the outset of her career; by her sagacity and good sense she immeasurably raised the position occupied by women in the general estimation, gave courage and enterprise to her sex, led the way for the rich to wisely utilise their wealth, during their lifetime, for the good of the community, and was in fact, as has been well said, not only the devoted and trusted friend of Queen Victoria, but her life will stand, next to that of Queen Victoria herself, in the Victorian era. Loving freedom, justice, purity and the joy of doing good, the Baroness Burdett-Coutts' example may well stimulate every woman, to quit herself worthily, and to do her utmost, always, to maintain the principles from which she derives her own strength. She was deservedly and widely popular and beloved. Throughout her career she avoided publicity, and so little is actually known of her many good works, that the obituary notices, which have appeared, reveal a paucity of information, which is the most striking testimony to her worth and methods, that could be forthcoming. The Baroness was a noble woman, for to her initiative and strength of character women owe much, which they have probably failed, as yet, to appreciate at its true value.

### The Care and Nursing of the Insane.

By Percy J. Baily, M.B., C.M.Edin., Medical Superintendent of Hanwell Asylum.

### II.—NURSING THE SICK.

(*Continued from page* 176.)

*Treatment of Bed-sores.*

In dealing with patients in whom bed-sores are likely to occur the nurse must be constantly on her guard to avoid all the preventable causes of these injuries. We shall therefore consider their treatment under two distinct headings: (1) How to prevent their occurrence; (2) how to hasten their healing after they have developed.

(1) *The Prevention of Bed-sores.*—(*a*) All parts of the patient which are subjected to pressure as he lies in bed, especially the buttocks and the region of the sacrum, and any portion of the skin which shows a tendency to redness must be kept scrupulously clean and dry. Great care should be shown in removing every trace of soap after washing, by the use of two basins and two flannels as already explained. The parts must be very thoroughly dried, but should not be rubbed with rough or hard towels, the drying being effected rather by dabbing movements than by rubbing. When the skin is obviously very tender lint or cotton wadding should be used rather than ordinary towelling. When as much moisture as possible has been removed by this means the part should be dabbed with a pledget of cotton-wool soaked in spirit, or the spirit may be rubbed over the part with the palm of the hand. The spirit abstracts the moisture that is left and afterwards rapidly evaporates leaving the skin perfectly dry. The part must then be well dusted with some dusting powder.

(*b*) The position of the patient as he lies in bed should be frequently changed. It is not necessary that the change in position should be very great, all that is required is just sufficient movement to ensure an alteration in the part which has been receiving the greatest amount of pressure.

(*c*) The under-sheet and the draw-sheet must be kept perfectly smooth and free from all rucks, and the accumulation of crumbs in the bed must be prevented.

(*d*) In the use of bed-pans all risk of bruising or scratching the skin must be avoided by carefully lifting the patient when the pan is placed under him and again when it is removed and the instructions for the after treatment of the parts already given should be followed.

(*e*) As soon as any portion of the skin becomes red and shows a tendency to bed-sore the patient should be immediately placed upon a water-bed. This is the only means whereby the pressure may be properly diffused over the whole surface of the patient's body which comes into contact with his support. If the old-fashioned but excellent form of tank water-bed be employed the use of any sort of mattress may be done away with. In such cases the tank should be nearly filled with water, otherwise there is a risk of the patient's body coming into contact with the bottom which would not only entirely do away with the object of the water-bed, but would put the patient in a position ten times worse than it was before his removal. The place of the mattress should be taken by several layers of blanket, and if the habits are faulty a mackintosh sheet should be placed between them. Besides the use of water-beds much may also be done to relieve pressure by water and air cushions, and padding by means of cotton-wool.

(*f*) The redness which always precedes and is a warning of the approaching bed-sore is due to the fact that the circulation of the part becomes stagnant and the veins congested. This may to some extent be overcome by gentle friction with the palm of the hand which stimulates the circulation. The hand should be lubricated with some kind of antiseptic ointment. The ointment has no direct influence in warding off the bed-sore, but the mere fact of its making the cuticle greasy increases the resistive powers of this layer of the skin against moisture and thereby acts beneficially when the patient suffers from incontinence of urine and is more or less continuously wet. For this reason the basis of the ointment should not be lanolin. This friction should be applied after the part has been washed and thoroughly dried with spirit and before the application of the dusting-powder.

(*g*) It has been recommended that where a bed-sore is threatening the skin should be hardened by a dilute solution (1 per cent.) of formal. Personally, I have no experience of this method, but I cannot help fearing that any hardening of the living tissues by this reagent must be obtained at the expense of its vitality and therefore do not favour its use. The application of spirit, as already suggested, has to a less degree some hardening effect upon the skin, but its use is not recommended on this account.

(*h*) The patient's body and bed-linen must be kept absolutely clean. Patients whose habits are faulty should be changed immediately they are found to be wet or dirty, and in dealing with such cases the nurse must be constantly on the watch by day as well as by night so as not to leave her patient in this condition. It frequently happens that the sheets and body-linen may become moistened by the profuse perspiration of the patient. When this is so it is equally necessary to change the patient at once. The use of a mackintosh sheet always interferes with the proper ventilation of the bed and so prevents the evaporation of the sweat which, accumulating around the patient, irritates and softens the skin and thus favours the formation of bed-sores. Hence the use of a mackintosh is to be avoided except where it is absolutely necessary.

(2) *Curative Treatment of Bed-sores.*—(*a*) When there is no slough and little or no discharge the part should be kept clean by frequent washing with some mild antiseptic lotion such as boracic lotion or a dilute (1 to 50 or 100) carbolic lotion and afterwards carefully dried and sprinkled with iodoform and dressed with dry iodoform gauze. If the discharge is more abundant the dressing must be moist and should consist of iodoform or cyanide gauze wetted with boracic lotion.

(b) When the formation of a slough cannot be prevented every means should be adopted to hasten its removal. The sore should then be dressed with moist boracic dressings which require to be very frequently changed. As the slough invariably becomes septic the smell is often most foul and penetrating. In such cases a charcoal poultice is most useful since it not only greatly lessens the foul odour, but also hastens the removal of the slough. When the slough has separated the sore is to be treated as an ordinary healing wound; moist antiseptic dressing should be applied, and these require to be frequently changed since the discharge is often most profuse. When the wound is very deep, or if it should have overhanging edges, it should be packed with iodoform or cyanide gauze.

### The Nurses' Clinic.

### FEMALE CATHETER CASES.

Every nurse must learn during her period of training how to pass the catheter on a female patient. She must also know from the doctor the times when a catheter ought to be passed, and the times when it ought not to be passed, the different kind of catheters used, and why. Unless under very special circumstances the doctor in charge leaves this part of the treatment entirely to the discretion of the nurse. Nurses in hospital will usually notice that they have more catheters to pass in the gynæcological wards than in any other.

It is necessary to pass the catheter in the following cases :—Immediately before all abdominal operations having to do with the uterine organs, immediately before all operations having to do with the bladder or ureters, in cases of retention of urine from whatever cause, and when specially ordered by the doctor, which may happen in the after nursing of particular abdominal or urinary fistula cases. In the latter cases the nurse is often ordered to catheterise the patient four hourly, or sometimes a catheter is fixed into the bladder at the operation, and the nurse must remove it to clean, at stated times, and again refix in position. In all ordinary cases unless a special order has been given the patient must be encouraged in every way to pass urine herself. Patients who have had hysterectomy, ovariotomy, perinæorrhaphy, or other uterine operations, often have some difficulty in passing urine for a few days after operation. This difficulty may arise either through paralysis of the sphincter muscle, for the time being, or a nervous hysterical state of the patient. In all cases of this kind the patient ought to be coaxed and encouraged in every way to pass urine herself, and failing this hot fomentations must be tried, applied over the pubes, or in abdominal cases over the vulva, before resorting to the catheter, as, if the catheter has once been passed it is often the beginning of many passings, as the patient begins to look on it as the only means of emptying the bladder whilst she is lying. I have heard a patient say, "Oh, you know I don't pass it myself, I need to have it taken from me!" so that it is always best in these cases for a nurse to use all her powers of persuasion before letting a patient get into that state. When necessary, unless specially ordered, the catheter should not be passed oftener than eight-hourly.

The most common sort of catheters in use are the glass, soft rubber and gum elastic. No. 8 to 10 for an ordinary adult patient. Glass catheters are the most general except in special cases; these cases being, perhaps, where a patient is delirious and it is best not to risk passing a glass instrument lest it get broken during the operation, and so injure the patient's urethra; or in cases where there may be any abnormal growth or tumour pressing on the bladder, and in such cases the danger is of making a false opening by using a glass catheter; hence the use of soft rubber or gum elastic.

Glass or rubber catheters may be sterilised by boiling and gum elastic by lying in perchloride of mercury 1-2000 for several minutes. In cases where there is a perinæum wound the nurse ought to fix a piece of rubber tubing to the end of the glass catheter used to save any urine dribbling back on the stitches.

To catheterise a patient the nurse must first sterilise her instrument, and put it in a vessel containing some warm antiseptic solution, boracic preferred, as if there be much catheterising it does not irritate the patient's urethra as some other antiseptics do. She must have another basin with some stronger antiseptic, also warm, and a few sterilised swabs of gauze or wool; a receiver for her swabs when used, and a urinal, or small vessel for the purpose.

After leaving all these things ready and the patient in position, the most usual being on her back with the knees drawn up, the nurse must scrub her hands thoroughly with soap and water, and disinfect them so as to render them surgically clean. She must then begin operations by sponging the labia and round about the urethra with the warm antiseptic solution prepared; this must be done thoroughly and conscientiously so as to be perfectly sure that no discharge whatever is left which may find its way with the catheter into the bladder. The nurse must remember all the time that the disease of cystitis is so very easily set up that she will be held responsible by her doctor for any carelessness in the performance of this duty. For my own part, I believe that where there is frequent catheterisation the patient is not unlikely to get cystitis, not through any carelessness on the part of the nurse, but from the constant passage of a foreign body into the bladder; yet this is only a reason why the nurse should be more particular than ever to be absolutely and scrupulously clean in all cases of this kind. The lips of the labia are then kept apart by the left hand, whilst the catheter is passed with the right, gently up into the bladder. No force whatever must be used. As soon as the urine begins to come freely the nurse must place her left hand over the region of the bladder and gently but firmly press so as to expel the contents and to make sure that the bladder is perfectly empty before withdrawing the catheter. In removing the catheter, if glass, the thumb of the right hand should be placed over the orifice, or if rubber the tube should be nipped in order to save any urine still contained in the tube being spilled. Before and after use a stream of water should be passed through the catheter from the eye downwards.

Some nurses believe in lubricating the catheter before passing, in this case sterilised vaseline, or oil, should be used. In this, as in all other branches of nursing, practice and absolute and surgical cleanliness are the chief things which lead to perfection.

### The Scottish Railway Accident.

### NURSING THE INJURED AT ARBROATH INFIRMARY.

The first announcement that a terrible railway disaster had occurred within sight of the hospital—for Elliot Station, though about a mile distant, can be seen on ordinary clear days from our windows—was brought by a messenger, who asked us to be ready to take in any cases that were sent along, adding that he believed that a large number of persons were killed or injured. We were just experiencing one of the fiercest snow-storms that this district has ever known. The gale which accompanied it wrought fearful havoc, with the result that not a single telephone-wire remained intact, and telegraphic communication was entirely cut off. We had thus to depend upon our own resources. There are forty beds in the hospital. Of these, thirty-one were occupied, leaving nine available, and eight patients were immediately sent home to make additional room. These are contained in four wards—one of which is situated on the ground floor, and three upstairs—on which floor is also the operating-room. Two of the wards upstairs are male wards, the others are for females, while the downstairs ward is usually occupied by women. This last, fortunately, was cleared for the Christmas entertainment, so that we had a ward with nine empty beds at our disposal.

The nursing staff consists of a matron, a sister, and five other nurses. The whole staff was at once informed of what had occurred, and instantly commenced to prepare for the reception and treatment of the injured. Bandages, splints, and other mechanical appliances likely to be required were got in readiness, all available bedding for making extra beds was brought out, and a plentiful supply of hot-water bottles filled ready for use. The operating-room was also prepared for use. These and other preparations were completed before the first case arrived, so that there was not the slightest confusion by the time the sufferers began to be brought in. All those injured had been placed in another train and taken to Arbroath Station, from which place they were conveyed to the hospital, some in the one ambulance waggon of which the town boasts, others in cabs, according to their injuries. One of the surgeons drove directly from Elliot to the hospital, so as to arrive before the patients, while the other three went on to Arbroath with the train, and each accompanied one or more patients from the station to the hospital. There were sixteen persons in all brought in.

The outstanding feature of these cases was that the great majority of them suffered from fracture of the lower limbs, and that most of those who died had severe injuries of the internal organs. Beds were found for all of them, and by eight o'clock every case had been attended to—that is to say, put to bed, had the clothing removed, and splints or dressing applied—though it was past six o'clock before they began to arrive. Of course, a great amount of work yet remained to be done. The nursing of so many cases, most of which would in ordinary circumstances have had a special nurse to itself, was a serious problem, and as all communication with Dundee—railway, telegraphic, or telephonic—was entirely cut off, no assistance could be obtained from that quarter. Fortunately, two nurses were secured in town—one a member of the Edinburgh Nurses' Co-operative Association, who was nursing a private case, and another who had received hospital training, but was now no longer in the professional ranks. These formed the entire nursing staff until late in the afternoon of the following day, when three nurses arrived from Dundee. All, however, worked with that spirit which only those who are truly devoted to their profession possess. During the night, or rather the early hours of the morning, no fewer than five patients passed away. Later on it was decided by the surgeons that the operation of suprapubic cystotomy could no longer be delayed in the case of a patient with a fractured pelvis, though in deference to his wishes attempts had been made to get a message through to Edinburgh or Dundee, summoning a specialist for that purpose. This, of course, entailed additional work on all the staff, but it was successfully accomplished before help arrived from outside. Altogether the staff has every reason to be satisfied with the good work done, and undoubtedly the incidents of that strenuous night's work will long remain fixed in the minds of those who took part in it.

### Our Christmas Distribution.

The following letters have been received by us from the recipient of parcels of garments which, through the kindness of our readers, we were able to distribute :—

The Matron of the Evelina Hospital for Sick Children, Southwark, S.E., writes : I beg to acknowledge with very many thanks the present of useful clothing.

The Matron of the West Ham and East London Hospital, Stratford, E., writes : The two parcels which you have so kindly sent to this hospital arrived safely. Will you please accept my very sincere thanks for the garments sent for our patients ? The parcels contained the most useful assortment of clothing, and I can readily tell you with what pleasure and delight each gift will be received. Personally, I feel, and I am sure other matrons do too, that the organisation of this "Distribution of Clothing" from the offices of The Hospital is a most magnificent work and charity.

The Secretary of the East London Hospital for Children, and Dispensary for Women, Shadwell, E., writes : I am desired by the board of management to tender you their best thanks for your very welcome present of clothing and cards, and to assure you that the gift is much appreciated.

The Matron of Whitechapel Infirmary, Vallance Road, writes : I have received a large parcel of good clothing from you to be given to the poor. I assure you it is a most acceptable Christmas gift, and I shall make it my business to see that deserving poor persons get it. It is extremely kind of you to have thought of us, and believe me such kindness is highly appreciated at this infirmary.

The Lady Superintendent of the East End Mothers' Lying-in Home, 394 and 396 Commercial Road, E., writes : Your annual expression of goodwill to our mothers and babies in the East End has duly arrived and was received with great pleasure. I beg to acknowledge the many warm garments so kindly sent, with most grateful thanks. The poor mothers here are in great need and are very thankful for any help they received and charmed to get a warm pretty garment for themselves and babies.

### Death in our Ranks.

We regret to announce the death of Miss D. M. Purser, a probationer at St. Bartholomew's Hospital on December 17 last. The cause of death was enteric fever and pneumonia, which she contracted during the discharge of her duties at the hospital. Miss Purser, who was 25 years of age, had previously had three years' training at the Cancer Hospital, Brompton. Her remains were interred at Highgate Cemetery on December 20.

### Christmas in the Hospitals.

BY OUR SPECIAL REPORTERS.

As each Christmas passes it is encouraging to observe that there is not the faintest indication of any flagging on the part of matrons, sisters, nurses, and others responsible for the provision of seasonable entertainments in their efforts to afford patients in the wards the maximum of pleasure. When the practice was first started it was contended that this form of contributing to the sum of human happiness under conditions of pain and suffering, would cease to be pursued with zest directly it had lost the charm of novelty. But as a matter of fact, familiarity with the modes of celebrating Christmas in hospital and infirmary, instead of breeding apathy, has tended to increase enthusiasm; and a perusal of the reports supplied by our special reporters of visits to leading charitable institutions last week will convince even the incredulous that age does not wither, nor custom stale, the infinite variety of voluntary caterers for the amusement of the large class so entirely dependent upon others for any amelioration of their hard lot. Of course there is scope for anxiety and patience as well as for resourcefulness and ingenuity in devising and preparing for the entertainments; and there are, perhaps, misgivings lest failure, rather than success, should be the ultimate result. But for these there is ample compensation in the intense satisfaction derived from the contemplation of the joy the poor folks, who, though separated by sickness from their relatives at the great home festival of the year, have their attention effectually diverted from their own condition, and are able to indulge in at least a measure of that mirth which is the prevailing atmosphere elsewhere. So long as hospitals themselves are a necessity, it may be hoped that Christmas festivities will be maintained with all the spirit, all the brightness, and all the good temper, which marked them in the closing days of 1906. If they are a cause of gratification to the patients, they are a source of happiness to those who plan them, because the primary object in view is the happiness of others.

### Guy's.

Christmas at Guy's was, as usual, a great success. Its reputation was maintained, and even excelled that of former years. Taking a walk round the wards on Christmas Eve while the finishing touches were being added, one was struck with the original designs and with the artistic arrangement and grouping of colours. The pale splendour of one ward, which earned it the epithet of "The Lane by Moonlight," was thrown into contrast by the Oriental gayness of its neighbours' illuminated chrysanthemums. In another ward one stepped immediately under the shelter of a lych gate, while the accident ward boasted of a diminutive "wayside inn" quaintly formed of screens with a thatched roof. The surroundings themselves were almost sufficient to impart a jovial Christmas spirit, but as the day dawned there came other surprises. A present for every patient, and then the roast beef and plum puddings held sway. Music from the pianos, kindly lent to the wards, was heard on every side, and the grand finale arrived as the residents, transformed into "The Niggers," tramped into each ward to crack their jokes and sing their funny songs, to the joy of the children and convalescents. While the wards were having a right good time, the out-patients' department was packed with its hundreds of small guests, who devoured cake and bread and jam with amazing rapidity. The "cinematograph" was a huge source of pleasure to the youngsters, who greeted each picture with their own quaint remarks. The large Christmas tree was next stripped of its trophies, and finally a "parcel for mother" was handed to each child as he or she reluctantly started for home. The side rooms were dreams of spring time, for one walked under the hanging flowers of laburnum and wistaria, and on into a bower of roses beyond where the tired waiters and visitors could refresh themselves with tea and its usual accompaniments. Not until the evening had well advanced did the strains of merriment die away, and then the glories of the decorations succumbed as the lights went out and the tired patients settled down to sleep and rest.

### The London.

The celebrations on Christmas Day at the London Hospital are distinguished by the excellence of their organisation; by the munificence of the gifts sent in from kind friends and sympathisers; and by the heartiness and goodwill with which all concerned do their utmost to promote the happiness and amusement of the inmates. The festivities begin and end on Christmas Day, so that they must necessarily start at an early hour, and in fact it was no more than four o'clock a.m. when a long procession of sisters and nurses, a hundred in all, paced solemnly into the wards bearing coloured lanterns in their hands and singing as they went some of the old, well-known carols. It took a long time to complete the rounds of all the wards, so that there was no very long interval between the departure of the carol-singers and the appearance of Father Christmas attended by two clowns and a bevy of fairies (little convalescent girls). On arriving at the wards Father Christmas found at each door a sack in which the sister had the presents for each patient wrapped up and directed. These presents are gifts from Sir Edgar and Lady Speyer and consist of a warm garment for each patient, such as a flannel shirt or petticoat, or knitted cardigan waistcoat. These were doled out by Father Christmas and distributed by his fairies. Nor were the sisters and nurses overlooked in this wholesale dispersal of presents, for there followed in Father Christmas's train a porter drawing a trolley piled high with fragrant violets, a gift from the resident medical officers to each sister and nurse in the hospital; a custom which started as long as twenty years ago. The next great event was dinner, and the thirty residents, appropriately attired as chefs, did the carving and had a very busy time of it. In the afternoon the men were allowed to smoke, and so ample were the gifts in this line sent by the chief firms of tobacconists, that each man received a new pipe, two ounces of tobacco, and a packet of cigarettes. At three o'clock began the entertainments, which went on without intermission until 7.30. Each entertainment lasted half an hour only, and each was given by a different troupe, causing the greatest possible amusement. At eight o'clock a dance for the wardmaids and scrubbers took place in the out-patients' hall, and they also got their share of the entertainments provided by the various troupes.

### Westminster.

Carols by the boys of the Westminster Hospital choir ushered in Christmas Day at the Westminster Hospital. Then followed a visit from Father Christmas, who kindly supplied a present for every patient. Dinner, for those well enough to enjoy it, consisted of turkey and plum pudding, while for less advanced patients there was fish and custards. After dinner the men patients received permission to smoke. Entertainments were also given by the resident medical staff and students, and caused much fun and merriment. Considerable interest is taken each year in the monster bran pies which here are a peculiar feature of

### CHRISTMAS IN THE HOSPITALS—*Continued.*

the Christmas Day festivities. Each sister has her own special bran pie, and ingenious brains and clever fingers are taxed to the utmost in order to contrive novelties every year. A huge plum pudding and an enormous bell, which descended to allow dips to be made in it, were among the most remarkable this year. One ward was decorated in Japanese style in honour of its bran pie, which took the shape of a gigantic Japanese lantern. A bran pie much appreciated was one made to resemble a portcullis, which, being the arms of Westminster was highly appropriate. But perhaps the most startling novelty, and certainly the most amusing, was the brain pie disguised as a savoy cabbage. Of course, the bran pies are all well stocked with presents, and the patients much enjoy the glorious uncertainty of a dip. For the children's ward a large Christmas tree, well laden with toys, was provided.

### St. Bartholomew's.

There was an excellent entertainment in the Grand Hall at St. Bartholomew's Hospital on December 31, when the performance of the farcial romance, "His Excellency the Governor," was given by the amateur dramatic club and the musical society attached to the institution. The considerable number of inmates present heartily enjoyed the programme, and were enthusiastic in their applause. The whole of the parts were admirably portrayed. Of the leading characters, Mr. S. S. Strahan was Mr. John Baverstock, the private secretary; Mr. A. C. Wilson, Captain Charles Carew, A.D.C.; Mr. Vernon Favell, his Excellency Sir Montague Martin; Mrs. Walter Wood, Stella de Gex; Mrs. Noble, Mrs. Wentworth Bolingbroke; Miss Mary Strahan, Ethel Carlton; and Mr. H. Scawen, the Right Hon. Henry Carlton, M.P. The rest of the parts were played by Messrs. W. B. Grandage (Captain Rivers), C. B. D. Butcher (Major Kildare), R. S. Townsend (a sentry), Patrick Black (butler), L. J. Burra (footman), and E. Hopper (native servant). Mr. Vernon was stage manager, and Mr. A. C. Wilson assisted. The contributions of the Orchestral Society were "Past and Present," "March of the Boyards," "Mignon," "Masquerade," and "L'Estudientina," and Mr. Edmund Maney conducted.

### King's College.

All the time-honoured customs and festivities were strictly adhered to at King's College Hospital this Christmas time. The nurses sang the old sweet carols, the usual festive fare was partaken of. On Boxing Day the patients' tea was celebrated and the choirboys from the Temple came and sang to the inmates. There was high revelry on Friday, December 28, when from ward to ward the resident staff and students made their way in various troupes, everywhere causing merriment and jollity by their amusing songs and performances. The festivities were wound up on December 31 by a Christmas-tree for the chilldren. This was preceded by a representation of Punch and Judy. When this was over tea was handed round, and thus reinforced after the exertions caused by so much laughter, the little ones were able to turn their attention to the splendours of the magnificent Christmas-tree and to delight in the treasures that were presently dispersed among them. The Ralli ward, where these festivities were held, was a marvel of artistic decoration, and the rows of Chinese lanterns shed a softened glow on the beautiful flowers. It was difficult to remember that one was in a hospital ward, so fairy-like were the surroundings and so bright the faces of the onlookers. The Santa Claus Society sent presents to some of the wards, and members of the Society came themselves to distribute them.

### Charing Cross.

A new departure was made at the Charing Cross Hospital in the matter of the decorations of the wards. These have gradually become such a tax upon the resources of the sisters and nurses, that it was decided this year to restrict all decorations to the tables and mantel-pieces and electric light and gas brackets. The cost of these for the whole of the hospital only amounted to £13, which was provided by the Board. The result of this innovation was a gain in every way. Attention was thus concentrated upon the softly-toned paper shades and the floral decorations in the centre and ends of the wards, and the sisters and nurses were relieved from a great strain upon their resources, and were also spared much physical exertion in the fixing up of the decorations, so that they were surprised to find themselves so much less tired this Christmas than ever before. On Christmas Day the first event was the singing of carols by the choir of St. Martin's Church. At four o'clock began the tea given by the sisters, to which each patient was allowed to invite two friends. Troupes of pierrots entertained the patients throughout the day, and the Ladies' Needlework Guild provided an article of clothing for every grown-up patient. The children were not forgotten either, for the Earl of Kilmorey had sent a huge collection of toys, and each child also received a toy from those sent by the proprietor of *Truth*. Nurses, past and present, took part in the festivities. On December 28 a splendid Christmas Tree was provided for all the little out-patients who had attended the hospital during the past year. The small guests were also provided with a good sit-down tea, while their parents and guardians partook of a buffet tea.

### The Middlesex.

Christmas Day began at the Middlesex Hospital with the presentation of gifts to the patients, most of which are sent by friends for this purpose, the remainder being provided from a special Christmas Tree Fund. Each of the adult patients received an article of warm clothing, or some such useful present, with, in addition, some tea and soap, whilst the children had toys, warm clothing, and soap. For dinner, in place of the usual hospital fare, turkeys and plum puddings were provided, and a good number of the visiting staff came round to assist in the arduous task of carving the turkeys, which in many cases they had themselves supplied. The wards were decorated after a more or less uniform system; the electric lights had coloured silk shades over them, whilst quantities of flowers and plants were tastefully arranged in different places, and the numerous fairly lights gave very pretty effects. From 2 p.m. to 4 p.m. the friends of the patients were admitted. The arrangements in the cancer wards were the same as in the general wards, and the nurses in these sang carols at 7.30 a.m. Celebrations of the Holy Communion were held in the chapel at 5.30, 6.30, and 7.30 for servants and nurses, and at 9.30 for night nurses and any patients able to attend. Morning service was held for the patients at 10 a.m. The nursing staff and servants of the hospital were not overlooked on Christmas Day, and all received useful presents and enjoyed a good Christmas dinner. On this day only they are allowed to circulate freely through the wards, which is a privilege always much appreciated. In Princess May and Percy wards there were Christmas trees which were dressed on Christmas Eve, but left untouched until the following Thursday and Friday, when the much-gazed-at treasures were at last bestowed upon the delighted children.

### St. Mary's.

On Christmas morning every patient in St. Mary's Hospital received a suitable present—the men shirts, the women articles of underclothing, and the children toys. Those of the male patients who were well enough were allowed to smoke. There were three celebrations of the Holy Communion in the chapel. The service at 11 a.m. was fully choral, "Star of the East" being well rendered by the choir of nurses. Instead of the sermon three carols were sung, and at the close of the service Stainer's seven-fold "Amen." The wards were all decorated with plenty of evergreens and some very beautiful flowers. The Manners ward looked most spring-like with lovely blendings of green and yellow, and the children's wards, Crawshay and De Hirsch, were particularly attractive. The children themselves, happily playing with their toys, were eager to show their new possessions to all visitors. Pianos were provided for every ward. The patients had visitors from three to four p.m.; then the great business of the evening, "The Ward Teas" began. The tables were artistically decorated with flowers and literally groaned under the weight of good things. The resident surgical, medical and obstetric officers worked hard to amuse the patients, and several of the nurses sang and played solos. The children's Christmas tree was held in the Board-room on Thursday, December 27.

### St. George's.

Christmas Day at St. George's Hospital was kept very quietly; the usual seasonable fare was provided, and the friends of the patients were allowed to visit them. On December 27 the customary Christmas tea was given, and the lady visitors, members of the Board, etc., attended in large numbers and added much to the enjoyment of the inmates, both by the gifts they so lavishly bestowed, and by the kindly chats they held with the patients. The little ones were rendered supremely happy by the stately Christmas-trees that delighted their dazzled eyes, and from which toys showered upon them in almost bewildering profusion. This hospital also came in for a share of the toys sent by *Truth*. On December 31 an entertainment, consisting mainly of a conjuring performance, took place in the board-room, and thither the patients were carried down and ranged round the room on their stretchers. It is an operation which occupies a considerable time and which requires no small amount of care and labour; indeed, an hour and a half is usually allotted to installing these helpless patients in their places. This year no fewer than 150 patients were able to be present.

### Royal Free.

It was a pleasant relief on Christmas Eve to exchange the slush of the street for the polished floors of the Royal Free Hospital. On that evening the season's festivities were already drawing to a climax, and every ward bore evidence of a quiet but happy expectation of what the morrow was to bring forth. Everywhere cheerfulness reigned. Each sister had arranged the decoration of her particular ward according to her own taste, and a most agreeable variety was the result—here a lavish display of flags, there a liberal distribution of evergreens, and elsewhere the soft, warm illumination obtained by an ingenious utilisation of Chinese lanterns. The monotony of hospital existence was broken to good purpose in the week before Christmas. A band of about thirty students went the round of the wards bearing Chinese lanterns on poles, singing carols and, more significant still, escorting Father Christmas, whose attendant satellites wheeled a stretcher laden with gifts. Each smiling patient received a parcel. Not content with this distribution the same ladies gave during the week two waxwork entertainments. Fittingly enough, however, Christmas Day witnessed the crowning fête. In the first place there was the usual relaxation of rule, and each patient enjoyed the privilege of inviting a friend to tea. Then a second distribution of parcels took place, this time at the instance of the authorities, and much excited, loosening of string and fumbling with brown paper was witnessed. It was the children, however, who proved the occasion for the supreme event of the Christmastide. In accordance with generous custom, Sister "Boys" gathered to her ward not only the juveniles at present in the institution, but those also who had been inmates during the year, and there, round a resplendent Christmas tree, a crowd of happy wee folk enjoyed a blissful hour rare enough in their straitened experience.

### Tottenham.

Christmas festivities at the Tottenham Hospital were brought to a close on Saturday evening last by a children's party. The function was held in one of the new wards which has not yet been opened; a spacious lofty hall, handsomely appointed. There were about 150 children present, including present and past patients, and their brothers and sisters. All the adult patients who were equal to it were also present, and a number of visitors, including many Governors. After a tea worthy of the occasion, and bonbons, a display of Maskelyne and Devant's living pictures was given. The display was of unusual excellence, and amused the children immensely. The proceedings were brought to a close in the good old-fashioned way. When the lantern sheet was removed a huge Christmas-tree was revealed, reaching from floor to ceiling, and richly laden with toys in great variety. The delight of the children knew no bounds, especially when Dr. Chappell, disguised as Father Christmas, proceeded to distribute the tree's burden. The party was organised by the matron, who was ably seconded by Mr. F. W. Drewett, the director, and backed up by the nurses. The entire cost of the party was collected by the matron, as usual, through the Christmas Tree and Entertainment Fund, which was well supported by the Governors and others with gifts in money and kind.

### Queen Charlotte's.

In a hospital like Queen Charlotte's little can be done for the entertainment of the patients, scattered about as they are in a number of small wards, and requiring quiet as part of their cure. The long corridors on each floor, however, were festooned with paper chains, and each ward boasted its sprig of holly. Christmas dinner must have been a formidable function for housekeeper and cook. At 9 a.m. the night nurses dined, and at one o'clock all the rest of the hospital staff, matron, sisters, doctors, students, and nurses —those in the hospital and the others who were doing their last month on the districts—a total altogether of seventy-eight. In spite of the fact that most of the nurses in this, as in other lying-in hospitals, are but birds of passage, the dinner was unmarred by any sense of estrangement. In a short time they might be scattered all over the world, never to meet again; but for this day and this hour they were bound by a common kinship, the memory of which would return to them—with the flush of their early ambitions and resolutions—in years to come. "Husbands' tea" in the wards followed as a natural sequence; and when the work of the day was done, the babies washed and fed, and the mothers comfortably settled, the nurses proceeded to keep up their good old custom of Christmas Night. Forming in a long procession, white-robed and white-capped, like a celestial choir, carrying coloured lanterns in their hands and singing carols, they went the rounds of the darkened wards. Perhaps the mothers, lying peacefully there and feeling the soft warmth of their tender babes beside them, could realise more than most people that wonderful Birth in Bethlehem 1900 years ago.

### CHRISTMAS IN THE HOSPITALS—*continued.*

### Samaritan Free.

A profusion of decoration distinguished the Samaritan Free Hospital, and testified to the ingenuity of the nurses. A portly Father Christmas, in snow-flecked garments, greeted the visitor inside the door, and none would guess that the red cloak covered an old sack stuffed with straw. The hall and staircase made a bower of evergreen, gleaming with fairy lamps and holly berries. Upstairs, on the first floor, the decorations had been carried out in Japanese style throughout. The archways were very prettily festooned with trailing twigs of green. In the centre of each was an inverted Japanese umbrella, from the spikes of which were suspended coloured lanterns, while a multitude of bells, silver and gold, of all sizes, hung from the glistening boughs. The wards made an equally tasteful display, with dado and frieze of white Japanese papers, and perpendicular lines of silver tinsel and green between the windows, the lamps softened with a variety of shades, while cleverly-fashioned paper butterflies perched here and there over the beds. Fortunately, although there were few empty beds, there were not many very bad cases, so most of the patients were able to avail themselves of the privilege of having their friends to tea on Christmas Day—and a merry tea-party it was.

### Hospital for Sick Children.

Every small inmate of the Hospital for Sick Children found, on awaking on Christmas Day, that Santa Claus had been on his rounds in the night and had left a stocking by the side of each cot, stuffed with the most desirable treasures. Carols having been sung by the nurses, a grotesque procession paraded through the wards to the great delight of the youngsters. It consisted of the residents got up in the most startling garbs, including, of course, Father Christmas and a plum pudding, as well as Mother Goose, a mastiff, and a lion. On December 27 the out-patients, to the number of 250, had their share in the Christmas festivities. The proceedings began with tea, after which there was a Punch and Judy show, followed by a distribution of presents from a mighty Christmas tree. December 28 was the great day for the in-patients, to which all the children looked forward with the utmost impatience, for on that day there was a most lovely tree set up in each ward, and every little boy and girl received so many really beautiful presents that they must have thought themselves transported to fairyland, where, as all children know, you have only to wish and you get all that you want. The Christmas trees were, of course, the centre of attraction in each ward, and several of them had some novelties to display. One tree had a number of birds perched on the branches, giving it a most realistic air, and in another ward a flight of swallows cunningly poised on an almost invisible wire appeared to be winging their way to the tree. Most of the trees shimmered with long threads of tinsel, resembling hoar-frost in the bright sunlight; and all of them glittered and shone with so brave a glory that it was no wonder that at first the little occupants of the cots sat and gazed at them in silent admiration, not unmixed with awe. Dolls and trumpets perched on the branches, and underneath each tree was a great pile of presents, books, and playthings of all kinds, which a little later were distributed among the eager, happy children. The beautiful flowers which decorated the tables in the centre of each ward afforded much scope for artistic arrangement, and most successful were the various achievements in this line. The paper shades for the electric lights added greatly to the effect, toning the scheme of table decoration. It was a wonderful day for the little sufferers and one that will form a bright spot in their memories for many a long year to come.

### East London Children's.

The committee of the East London Hospital for Children believe in keeping Noel with an unstinted holiday. All the forces were concentrated on Saturday's celebration. Chinese lanterns and a charming arrangement of evergreens greeted the visitor on crossing the threshold to tell of the festivities afoot. It was a promise of gaiety well borne out. The hub and centre of it all was the out-patients' hall. There, after memorable tea parties in the five wards, the majority of the little folks were assembled. It was a scene to remember, this gathering in the spacious hall whose walls, brightly decorated that day, must witness many a tragic incident in the drama of hospital life. A screen raised at one end served the double purpose of displaying cinematograph pictures and concealing a mystery whose final unveiling was to be the climax of the occasion. Squeezed into a little room at the side, and, indeed, overflowing it, a dozen bandsmen of the 2nd Life Guards discoursed the brightest of music, and incidentally made a brave show in their scarlet uniforms. There were other scarlet uniforms in the room. The wearers, little soldiers of suffering, laid on mattresses or seated in chairs, had the places of honour among the audience. It was an appreciative audience, too, and the doctors, nurses, students, and visitors enjoyed everything almost as much as the little patients themselves. The moment for revealing the secret brought with it an unrehearsed surprise. Hardly had the sheet been lowered, disclosing for one breathless moment a pair of gigantic Christmas trees, bright with toys and sparkling with lights, when all at once the room was plunged in darkness. But this was speedily remedied, and soon Father Christmas stepped forward and began to distribute his treasures, the wee folks' happiness at this stage reaching a point beyond expression. Among those who contributed to the success of the festival by gifts of toys and clothing were the Princess of Wales and her children.

### Paddington Green Children's.

On Christmas Eve each little patient in Paddington Green Children's Hospital hung up the bag which was provided instead of a stocking on the end of its cot, in simple happy trust of what the mysterious night would bring forth; and Santa Claus, in the person of Miss McGregor, the matron, duly filled it up with toys and gifts from nurses and unknown friends. Then, two days later, came the entertainment. The glories of the Christmas tree, illuminated with numberless tiny lamps and electric light, and laden with toys, were partially visible through the windows even to the passers-by outside. There were toys for everyone—such as many of these poor little mites had never dreamed of possessing. In the upper, or surgical, ward the cots were all pushed up to the top, close together, where the little occupants could have the best view of the magic-lantern pictures kindly shown by Dr. Herbert E. Friend; and all the medical patients who could possibly be moved were carried up and placed in chairs in the front. An excellent collection of slides was exhibited. "One of our Japanese Allies" was thrown on the screen—a little Japanese child who had inhabited one of the cots—and the greeting to the matron and nurses was received with warm applause. When a placard on the screen announced "The End," the little ones sat still in their chairs, patient and hopeful; and even when a few bars of God Save the King were played on the piano no one budged till the lights were turned on, and the nurses picked up the tiny ones and carried them off to bed. Two of them had already fallen asleep in their cots. In the Board-room tea was dispensed by some of the sisters to the numerous visitors.

### Everybody's Opinion.

[Correspondence on all subjects is invited, but we cannot in any way be responsible for the opinions expressed by our correspondents. No communication can be entertained if the name and address of the correspondent are not given as a guarantee of good faith, but not necessarily for publication. All correspondents should write on one side of the paper only.]

### NURSES AND THEIR HONORARY PHYSICIAN.

Dr. Washington Isaac, 75 Gower Street, W.C., writes: I wish as late honorary physician of the Nurses' Hostel, through your columns, to thank the eighty-seven nurses of the Nurses' Hostel, North and South blocks, and the Nurses' Lodge, Colosseum Terrace (late of the Nurses' Hostel) for the silver rose-bowl presented to me on Christmas Day as a token of their appreciation of my services whilst I was their honorary physician.

### CHRISTMAS PRESENTS—A SUGGESTION.

"District Nurse" writes: Would not the question of Christmas and other presents be solved by abolishing the subscription lists and letting everybody have full liberty as regards the giving a present or not? It will be argued that, by collecting a large sum, a more costly gift than would otherwise be possible, can be purchased. But I take it that the value of a gift does not consist in the cash it represents, but in the motive which prompts the giver. Present-making should be an entirely individual matter, because only then can the recipient be sure of the sincerity of the act. When it comes, however, to a community of persons, where each is expected to give his or her share, we may be pretty certain, barring exceptions, of course, that a few of them will act on compulsion, and, as far as they are concerned, the gift is not a gift—an expression of goodwill—at all.

### Appointments.

[No charge is made for announcements under this head, and we are always glad to receive and publish appointments. The information, to insure accuracy, should be sent from the nurses themselves, and we cannot undertake to correct official announcements which may happen to be inaccurate. It is essential that in all cases the school of training should be given.]

Belfast Hospital for Sick Children.—Miss Constance Rome has been appointed lady superintendent. She was trained at Addenbrooke's Hospital, Cambridge, and has since been theatre sister and night superintendent at Poplar Hospital, sister at Nottingham Children's Hospital, sister at Manchester Children's Hospital, assistant matron at the Cosmopolitan Hospital, Venice, and sister at the East London Children's Hospital, Shadwell.

Birkenhead Union Hospital and Infirmary.—Miss Harriet Copeland has been appointed assistant superintendent of nurses and house sister. She was trained at Birkenhead Union Infirmary, where she has since been charge nurse.

Birmingham General Hospital.—Miss J. E. Pritchard has been appointed sister. She was trained at St. Bartholomew's Hospital, London, and has since been a sister of the Army Nursing Service Reserve.

Bradford Children's Hospital.—Miss E. Johnstone has been appointed sister. She was trained at Sunderland Infirmary, where she has since been charge nurse.

Cottage Hospital, South Wimbledon.—Miss Phillis J. Dawson has been appointed staff nurse. She was trained at Guy's Hospital, London, and has since been nurse at Dr. Barnardo's Hospital for Sick Children. She has also done private nursing.

Cronley Sanatorium, Kingswood, Frodsham.—Miss Marie Moore has been appointed charge nurse. She was trained at Mount Vernon Hospital, Hampstead, where she has since been assistant nurse. She has also been charge nurse at Crooksbury Sanatorium, Farnham.

Lambeth Infirmary, Brook Street, Kennington Road, S.E.—Miss Beatrice Selina Dittmer and Miss Ethel Green have been appointed ward sisters. Miss Dittmer was trained at the Grantham Hospital and at King's College Hospital, and has since held the post of sister at the country branch of the Royal Hospital for Sick Children, Glasgow. Miss Green was trained at Lambeth Poor-law Infirmary, and has since done private nursing. She is qualified in massage.

Manchester Royal Eye Hospital.—Miss L. M. Candy has been appointed sister. She was trained at the General Infirmary and Eye Institution, Gloucester. She has since been in charge of a ward in the same institution, and sister at North Staffordshire Infirmary and Eye Hospital, Hartshill, Stoke-on-Trent.

New Zealand.—Miss Hester McLean has been appointed assistant inspector of the hospitals. She was trained at the Alfred Hospital, Sydney, and has been matron of the Women's Hospital, Melbourne, and sister of the District Nursing Association in Melbourne and Sydney.

Rotherham Hospital and Dispensary.—Miss Ethel Towers Minors has been appointed night superintendent. She was trained at Walsall General Hospital, and has since been sister at the Royal Hospital, Sheffield, and night sister at the Beckett Hospital, Barnsley.

Roxburgh District Asylum, Melrose.—Miss Blanche Ellis has been appointed sister in charge of the Male Infirmary. She was trained at Withington Infirmary, Manchester, and has since been nurse at the Cancer Hospital, Manchester, and sister at the Southern Hospital, Manchester. She has also done private and district nursing.

Wolverhampton Workhouse Infirmary.—Miss Ada Paule has been appointed charge nurse. She was trained at Leeds General Infirmary, and has since been ward sister at Sheffield Royal Infirmary, and midwife attached to the Cheltenham District Nursing Association.

Workhouse Infirmary, Smithdown Road, Liverpool.—Miss Margaret Ann Blackclock has been appointed charge nurse. She was trained at Sheffield Union Infirmary, and has since been staff nurse at Firvale Infirmary, Sheffield.

York County Hospital.—Miss Evelyne Osborne has been appointed sister. She was trained at the Rotherham Hospital and Dispensary, where she has since been staff nurse and taken temporary sister's duty.

### Presentations.

Belper Union Workhouse.—On Friday, December 28, Miss F. Brotherton, the retiring superintendent nurse, was presented with a silver-mounted writing cabinet by the staff.

Brighouse Joint Hospital.—The members of the Board of Brighouse Joint Hospital presented to Miss Waddington, the matron, on her retirement in order to be married, a silver tea service and an illuminated address, containing the signatures of the Board and of the officials of the Corporation.

Manchester Royal Eye Hospital.—Sister Edgar, on leaving the Manchester Royal Eye Hospital to take up duty at Southampton, was presented by the nursing staff with a handsome cabin trunk and week-end bag, and by her patients (past and present) with a writing case and a Swan pen.

### Notes and Queries.

**REGULATIONS.**

**The Editor is always willing to answer in this column, without any fee, all reasonable questions, as soon as possible.**

**But the following rules must be carefully observed.**

**1. Every communication must be accompanied by the name and address of the writer.**

**2. The question must always bear upon nursing, directly or indirectly.**

**If an answer is required by letter a fee of half-a-crown must be enclosed with the note containing the inquiry.**

*Dispensing.*

(156) Can you tell me where I can learn dispensing?—*M. J.*

Write to the Pharmaceutical Society, 17 Bloomsbury Square, W.C., and ask for advice.

*Training.*

(157) I have not finished my training in London. Can I count the period of training I have received in continuing at a provincial hospital?—*North.*

No. You must train in one institution for the full time in order to secure a certificate.

*Deaf and Dumb.*

(158) Will you kindly let me know if there is any institution which would receive a deaf and dumb woman of 30?—*District Nurse.*

The British Asylum for Deaf and Dumb Females, Lower Clapton, E., receives women up to 30 years. You might write to the Office, 5 Bloomsbury Square, W.C., and ask what they can do for you.

*Sanitary Inspector.*

(159) I wish to become a lady health inspector. Can you tell me what is the proper course of training to qualify?—*Lina.*

Write to the Secretary of the Royal Sanitary Institute, Margaret Street, W.

*Poor-law Superannuation Fund.*

(160) Is it possible to join the Poor-law superannuation fund after contracting out as a probationer?—*Birkenhead.*

The Secretary, Local Government Board, Whitehall, S.W., may be able to tell you.

*Probationers with Salary.*

(161) Will you kindly tell me if there is any hospital or institution in or around Manchester where they take probationers with small salary, and who shall I write to?—*Anxious.*

The Manchester Royal Infirmary gives £10 for the first year; the Crumpsall Infirmary and the Chorlton Union Infirmary, West Didsbury, also give salaries. Write to the lady superintendent.

*Nursing in India.*

(162) Can you tell me where to apply for information concerning Lady Minto's Indian Nursing Association?—*Liverpool.*

Write to the Hon. Secretary of the Indian Nursing Association, Simla, India.

*Kleptomania.*

(163) Can you help me to find a home where control would be exercised over a child of 12 who has kleptomania?—*Manchester.*

The case would seem to be one for a school of the feeble-minded, such as Adcote, Knotty Ash, Liverpool. The charge is 6s. per week. Write to Mrs. H. Pilkington, Wheatshill, Huyton, Liverpool.

*Champagne Cork.*

(164) Can anyone recommend a champagne cork which will allow a small quantity of wine to be poured out at a time?—*Huddersfield.*

We have made enquiries and cannot hear of any but the ordinary one used at refreshment-rooms mainly, which we conclude is the one you do not wish for. There is a very secure rubber cork, but this has a screw and closes by leverage, and the bottle must be opened entirely each time. The other permits of a small quantity being extracted without opening each time. Any large store or ironmonger would have it.

**Handbooks for Nurses.**

| | Post Free. |
|---|---|
| "How to Become a Nurse: How and Where to Train." | 2s. 4d. |
| "Nursing: its Theory and Practice." (Lewis.) ... | 3s. 6d. |
| "Nurses' Pronouncing Dictionary of Medical Terms." | 2s. 6d. |
| "Complete Handbook of Midwifery." (Watson.) ... | 6s. 4d. |
| "Preparation for Operation in Private Houses." ... | 0s. 6d. |

Of all booksellers or of The Scientific Press, Limited, 28 & 29 Southampton Street, Strand, London, W.C.

### For Reading to the Sick.

FEAR NOT, I AM WITH THEE.

Father, I do not ask
That Thou wilt choose some other task
And make it mine. I pray
But this: Let every day
Be moulded still
By Thine own hand; my will
Be only Thine, however deep
I have to bend Thy hand to keep.
Let me not simply do, but be content,
Sure that the little crosses each are sent;
And no mistake can ever be
With Thine own hand to choose for me.—*Anon.*

There is no true rest of soul and heart in time of trial, until we have come to look beyond second causes and human instruments, and have seen the Hand of our God and Father appointing all in His infinite wisdom and love. Cease as far as possible from vain regrets, and leave all calmly to Him, Who has promised that all things shall work together for good. To bring our hearts to this may be the very meaning and end of the dispensation.—*Anon.*

Surely in our saddest moments of loneliness and desolation, if our eyes were opened, we should see friends and comforters; angels; spirits of blessed ones who have known sorrow, but know it no more; our own beloved who have passed over Jordan, and can now witness our trials, with the glad certainty that these are but bringing us nearer the Haven they have reached, and we would fain reach,—shall reach, by God's grace, in a little while;—all these, and countless hosts of the Great Invisible World which wraps us round, we should see, were it granted us. But sight—such sight—might make the yearning too intense for us to persevere our appointed time here, and therefore we must rest on faith a little while. Only believe that your heart's need of love is known to your Father,—is it not He Who has made you so?—and in a way you know not, think not of, He is leading you closer and closer to Himself by means of it. Somewhat of loneliness in life, a passage thence which no earthly love can share,—"je mourrai seul," as Pascal says—and then—oh, then, what fullness of joy and love and endless satisfaction for evermore! Cannot we wait in patience a little while, with such certainty before us?

*Sidney Lear.*

SAY, is the child alone,
Whose hand the Father holds;
Or whom, unseen but not unknown,
The Friend of friends enfolds?

And still, in calm or storm,
In throngs or desert rude,
Beside thee moves His radiant Form,
Is this thy solitude?

At daybreak He is there,
With healing in His wings,
And in the quiet midnight air,
The balm of Gilead brings.

He calls me to His throne;
I go with Him to be;
And lonely, thou art not alone,
While He abides with thee.

*Bishop Bickersteth.*

